# Long-term survival benefit from third-line treatment for advanced colon cancer: a case report

**DOI:** 10.3389/fimmu.2025.1639287

**Published:** 2025-09-12

**Authors:** Ju Su, Yuqing Kuang, Yiwen Wu, Qun Chen, Shadong Min

**Affiliations:** ^1^ Department of Oncology, People’s Hospital of Xiangxi Autonomous Prefecture/The First Affiliated Hospital of Jishou University, Jishou, China; ^2^ Department of Pharmacy, People’s Hospital of Xiangxi Autonomous Prefecture/The First Affiliated Hospital of Jishou University, Jishou, China; ^3^ Department of Hematology, People’s Hospital of Xiangxi Autonomous Prefecture/The First Affiliated Hospital of Jishou University, Jishou, China

**Keywords:** colorectal cancer, liver metastasis, sintilimab, fruquintinib, bullous pemphigoid

## Abstract

**Background:**

Colorectal cancer (CRC) is a common malignancy with a relatively high incidence and mortality rate. Treatment options for metastatic CRC (mCRC) in the third-line setting remain limited. This article reports a case of colon cancer with liver metastases that progressed after comprehensive treatment but achieved a prolonged progression-free survival of over 38 months with third-line immunotherapy combined with targeted therapy. This aim is to improve understanding of CRC with liver metastasis and provide a clinical reference for managing similar cases.

**Case presentation:**

A 69-year-old male presented to the People’s Hospital of Xiangxi Autonomous Prefecture in April 2021 with a 2-week history of fatigue and abdominal distension. His medical history included type 2 diabetes mellitus and diabetic peripheral neuropathy, managed with long-term insulin therapy. Based on clinical presentation, imaging studies, gastroscopy and colonoscopy, and pathological findings, he was diagnosed with moderately to poorly differentiated adenocarcinoma of the ascending colon with liver metastasis (cT4aN0M1a IVA, proficient mismatch repair [pMMR], clinical risk score [CRS] 2). The patient received 6 cycles of neoadjuvant chemotherapy with oxaliplatin, calcium folinate, and fluorouracil (the first 3 cycles combined with bevacizumab), followed by radical resection of the primary tumor and resection of complex liver metastases. Postoperatively, he underwent five cycles of adjuvant chemotherapy with oxaliplatin, calcium folinate, and fluorouracil (the last four cycles combined with bevacizumab). In November 2021, bilateral lung metastases were detected. The patient then received three cycles of chemotherapy with fluorouracil, calcium folinate, and irinotecan, but the disease continued to progress. From February 2022 to April 2025, third-line treatment with fruquintinib (targeted therapy) combined with sintilimab (immunotherapy) was initiated.

**Conclusion:**

Fruquintinib combined with sintilimab immunotherapy may represent a promising third-line treatment option for patients with pMMR mCRC.

## Introduction

Colorectal cancer (CRC) ranks as the third most commonly diagnosed cancer and the second leading cause of cancer-related death worldwide (1). Standard treatment strategies include surgery combined with chemotherapy, radiotherapy, or targeted therapy, etc. However, therapeutic outcomes remain unsatisfactory for many patients, especially those with advanced disease who are prone to recurrence and metastasis. The liver is the most frequent site of distant metastasis in CRC, with approximately 50% of patients developing liver metastases over the course of the disease, and nearly half of these are detected at initial diagnosis (2). Liver metastasis of CRC may be attributed to several factors: the portal venous system facilitates hematogenous dissemination of tumor cells from the colon to the liver; and the hepatic microenvironment provides an optimal niche for metastatic colonization (3). Liver involvement significantly impacts prognosis. Therapeutic options become increasingly limited in the third-line setting. Fruquintinib, a targeted agent commonly used at this stage, provides a median overall survival (OS) of only 9.3 months, whereas combining fruquintinib with the PD-1 inhibitor sintilimab has been reported to extend median OS to around 20 months. Here, we report a case of colon cancer initially diagnosed with synchronous liver metastasis. After successful conversion therapy and surgical resection, the patient developed multiple lung metastases within 3 months postoperatively and experienced progression again 2 months after second-line treatment. Third-line therapy comprising sintilimab and fruquintinib was initiated, during which the patient developed bullous pemphigoid as an immune-related adverse event (irAE) but improved with hormone therapy and was able to continue treatment. Remarkably, a progression-free survival (PFS) of more than 38 months was achieved. The patient described in this case report exhibited a PFS far longer than that typically observed in other advanced colon cancer patients receiving third-line therapy, representing a particularly notable phenomenon. The aim of this case report is to enhance clinicians’ understanding of metastatic colon cancer, to improve recognition and management of sintilimab-related adverse reactions, to contribute to the knowledge base on immune checkpoint inhibitor (ICI) toxicities, and to provide a clinical reference for the treatment of similar patients.

## Case presentation

**Figure 1:**
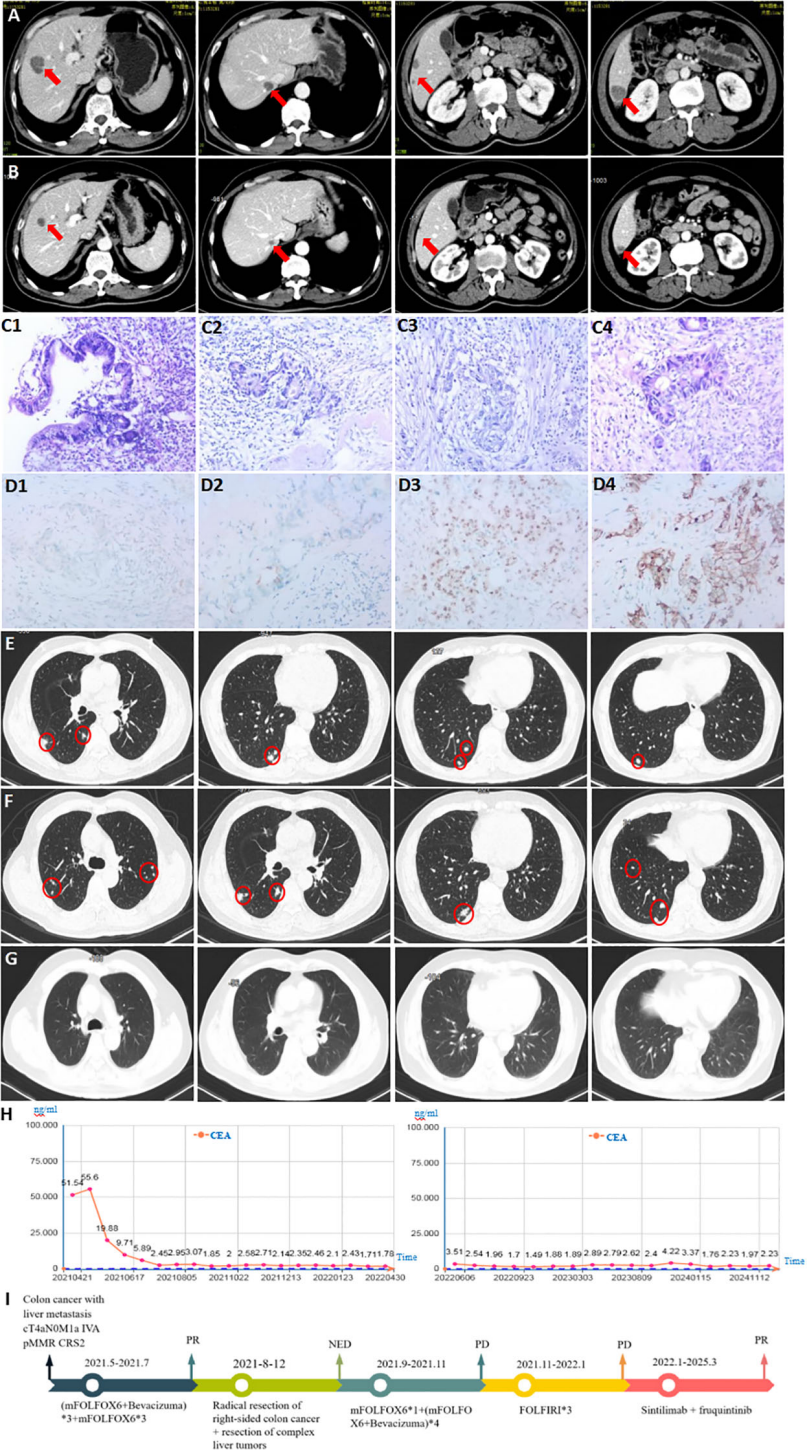
Description of patient imaging findings and clinical management. **(A)** Liver metastatic lesions of the patient before the initial treatment; **(B)** Liver metastatic lesions of the patient after 6 cycles of chemotherapy and 3 cycles of targeted therapy, with therapeutic effect of PR; **(C)** Postoperative histopathological features (H&E, ×20): 1) Colon tissue, 2) The S6 segment of the liver, 3) The S7 segment of the liver, 4) The S8 segment of the liver; **(D)** Immunohistochemical profile (H&E, ×20): 1) Complete loss of CK7 expression in tumor cells, 2) Focal expression of CK20, 3) Positive expression of CDX2, 4) Positive expression of Villin; **(E)** Multiple pulmonary metastases occurred after the patient’s surgery (November 2021); **(F)** Therapeutic effect after 3 cycles of chemotherapy for pulmonary metastases, which was PD; **(G)** Last re-examination of the patient after third-line targeted and immunotherapy (March 2025); **(H)** Trend chart of CEA Variation; **(I)** Treatment timeline of the patient’s clinical course.

In April 2021, a 69-year-old male presented to our hospital with a half-month history of fatigue and abdominal distension. His past medical history was significant for type 2 diabetes mellitus and diabetic peripheral neuropathy, with long-term insulin use for glycemic control. After a comprehensive clinical evaluation, he was diagnosed with moderately to poorly differentiated adenocarcinoma of the ascending colon with liver metastasis (cT4aN0M1a stage IVA, pMMR, clinical risk score [CRS] 2) (**Figure 1A**). Differential diagnosis of liver lesions included: 1) Primary hepatocellular carcinoma (HCC): This is the most common primary malignant liver tumor, often associated with cirrhosis. On computed tomography (CT) imaging, HCC typically appears as low-density lesions on plain scans, shows significant enhancement in the arterial phase, and presents hypo-dense during the portal venous and delayed phases. 2) Hepatic hemangioma: This is a benign vascular tumor, usually cavernous. On non-contrast CT, lesions appear as uniform low-density masses, with characteristic nodular peripheral enhancement on contrast-enhanced scans.

**Figure 2:**
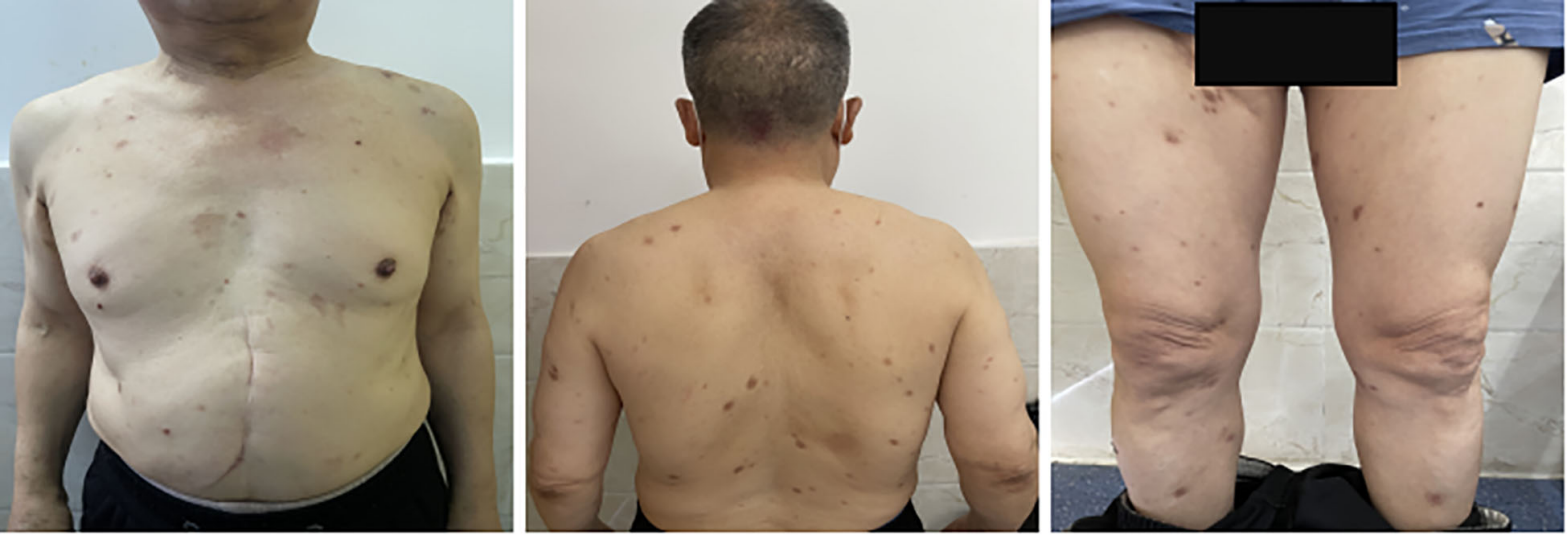
The patient’s bullous pemphigoid condition improved after treatment.

From May 1 to May 31, 2021, the patient received three cycles of FOLFOX6 (oxaliplatin + calcium folinate + fluorouracil) combined with bevacizumab. No obvious toxicities were noted, and the therapeutic response was evaluated as a partial response (PR). After discussion by a multidisciplinary team (MDT) and comprehensive assessment, disease control was deemed sufficient to proceed with conversion therapy; bevacizumab was discontinued preoperatively as per routine practice. From June 17 to July 18, 2021, he received three additional cycles of FOLFOX6. During treatment, only grade I gastrointestinal adverse events occurred, and efficacy remained PR (**Figure 1B**). On August 12, 2021, the patient underwent laparoscopic-assisted right hemicolectomy, complex liver tumor resection, and lysis of intestinal adhesions under general anesthesia. Postoperative pathology demonstrated: 1) Colon specimen—a few superficial epithelial cells of the nodular mass showed high-grade intraepithelial neoplasia with superficial erosion, consistent with post-chemotherapy changes (**Figure 1C1**). No tumor was seen in the submucosa, muscularis, or serosa. Surgical margins of the proximal and distal resection sites and omentum were negative, and no metastatic cancer was found in mesenteric lymph nodes (0/13), although one showed post-chemotherapy changes. 2) Liver—necrosis consistent with post-chemotherapy changes was observed in segments S6, S7, and S8; small residual tumor components were present in the second focus of S6, as well as in S7 and S8 (**Figure 1C2-3**). Immunohistochemistry showed CK7 (-), CK20 (focal +), Villin (+), CDX2 (+), and MUC-1 (-) (**Figure 1D**). Postoperative pathological stage was ypTisN0M1a (stage IVA), with a tumor regression grade of 0-1. Postoperatively, one cycle of FOLFOX6 and four cycles of FOLFOX6 combined with bevacizumab were administered. Only grade I gastrointestinal toxicity occurred. On November 20, 2021, the efficacy evaluation indicated progressive disease (PD). No abdominal abnormalities were seen on CT, but chest CT and PET-CT revealed multiple pulmonary nodules in the upper and lower lobes of the right lung and the lower lobe of the left lung, suggestive of metastases (**Figure 1E**). From November 29, 2021, to January 11, 2022, the patient received three cycles of FOLFIRI (fluorouracil + irinotecan + calcium folinate). Due to a heterozygous UGT1A1 mutation, the dose of irinotecan was reduced. During treatment, he experienced grade II gastrointestinal reactions and grade II myelosuppression. The therapeutic response was evaluated as PD. CT on January 24, 2022, again showed no abdominal abnormalities, but progression of multiple pulmonary nodules (**Figure 1F**). From February 2022 to March 2025, third-line treatment with fruquintinib (starting at 5 mg, later reduced to 3 mg) combined with sintilimab (200 mg every three weeks from March 2022 to March 2024, then every three months from July 2024 to March 2025) was initiated. During this therapy, grade III hand-foot syndrome and hypertension occurred. In September 2023, multiple blisters appeared on the skin and in the oral cavity, with some areas ulcerated. The BP180 antibody was positive; dermatology consultation diagnosed bullous pemphigoid. Immunotherapy and targeted therapy were suspended, and hormone treatment was initiated. After 2 months and clinical improvement, oral methylprednisolone maintenance was commenced, and combination therapy was resumed in November 2023 (**Figure 2**). The response was evaluated as PR, and the most recent follow-up on March 12, 2025, showed a decrease in the sizes of multiple pulmonary nodules (**Figure 1G**). The trend chart of carcinoembryonic antigen (CEA) is presented in **Figure 1H**. The full treatment timeline is outlined in **Figure 1I**.

## Discussion

The liver is the most frequent site of hematogenous metastasis in CRC, with 15%-25% of patients presenting with liver metastases at initial diagnosis. Although chemotherapy and targeted therapy can improve survival in selected individuals, only 20%–30% of patients with colorectal liver metastases are initially considered candidates for radical resection. Even among those who undergo surgery, 50%-60% experience recurrence. In patients whose liver metastases can be completely resected or who achieve a no evidence of disease (NED) status, the median survival is approximately 35 months, and the 5-year survival rate is 30%-57% (4). In the present case, conversion therapy allowed successful surgical resection and attainment of NED; however, this status persisted for only six months before multiple pulmonary metastases developed, and the disease progressed again.

Treatment strategies for patients with advanced CRC have become increasingly diversified, especially for patients with chemotherapy-resistant or metastatic diseases. However, the median OS for single-agent third-line therapies (ie, regorafenib, fruquintinib, trifluridine/tipiracil) ranges from only 7.8-9.3 months, with a median PFS of just 2.0-3.7 months (5–7). Fruquintinib is a highly selective oral inhibitor of vascular endothelial growth factor receptors (VEGFR) -1, -2, and -3, which suppresses tumor angiogenesis. In the CONCUR, TERRA, and FRESCO trials, third-line agents significantly prolonged OS compared with control; regorafenib and fruquintinib improved OS by 2.5 months and 2.7 months, respectively, while TAS-102 prolonged it by only 0.7 months. Similarly, median PFS was extended by 1.5 months with regorafenib and 1.7 months with fruquintinib, compared with 0.2 months with TAS-102 (5–7). Regarding safety, fruquintinib demonstrated the lowest incidence of grade ≥3 treatment-related adverse events among these agents. The frequency of severe hand-foot skin reactions with fruquintinib was markedly lower than with regorafenib, whereas hypertension could be effectively managed with monitoring and appropriate intervention (5–7). Among them, fruquintinib is an important third-line treatment option, with relatively superior efficacy. Although combining TAS-102 with bevacizumab can extend OS to 10.8 months, up to 70% of patients require treatment delays due to toxicity (8). Patients with pMMR/microsatellite stable (MSS) CRC exhibit an immunologically “cold” tumor microenvironment—characterized by low tumor mutation burden (TMB), scarce effector T cell infiltration, and enrichment of immunosuppressive cells—which contributes to poor response rates to ICIs as monotherapy. Anti-angiogenic therapy has been shown to normalize abnormal tumor vasculature, thereby enhancing T-cell infiltration, reducing suppressive immune cell recruitment, and downregulating hypoxia-mediated inhibitory signals such as PD-L1. In addition, VEGF blockade can prevent the compensatory upregulation of multiple immune checkpoints, conferring synergistic anti-tumor effects when combined with ICIs (9). In the REGONIVO study, regorafenib combined with nivolumab achieved a 33% objective response rate (ORR) and median PFS of 7.9 months in refractory MSS mCRC (10). Fruquintinib plus sintilimab also showed promising activity, with an ORR of 15.38% and median PFS of 3.6 months in one real-world cohort of 52 patients (11). A phase Ib study presented at the 2021 ASCO meeting further demonstrated that fruquintinib combined with sintilimab in pMMR/MSS mCRC achieved a median OS of 20 months, median PFS of 6.9 months, and a disease control rate (DCR) of 100% (12). Moreover, the randomized phase II CAPability-01 trial suggested that the combination of sintilimab, chidamide, and bevacizumab represents another promising strategy for pMMR/MSS mCRC (9). In the present case, third-line therapy with fruquintinib plus sintilimab yielded exceptional results. As of April 2025, the patient’s PFS has reached 38 months, and OS has exceeded 48 months, with ongoing clinical benefit.

In addition, the toxicities associated with immunotherapy require close attention. In this case, the patient developed cutaneous irAEs, the most common class of toxicities associated with ICIs, typically manifesting as rash, pruritus, or vitiligo, etc. (13–15). These reactions usually arise early during treatment but may also present several months afterward. Interestingly, some studies suggest that cutaneous irAEs may correlate with improved antitumor efficacy of PD-1 inhibitors; for example, vitiligo in melanoma patients has been associated with clinical benefit (16–18), a phenomenon also observed in renal cell carcinoma (19) and non-small cell lung cancer (20). Immune-mediated bullous dermatoses are less common, with a reported incidence of 1%–5%. They have been described in patients with melanoma, non-small cell lung cancer, CRC, and renal cell carcinoma receiving immunotherapy (20–24). Bullous pemphigoid is a rare autoimmune blistering disease caused by autoantibodies targeting BP180 and BP230 within the basement membrane zone, resulting in epidermal-dermal separation. Direct immunofluorescence typically demonstrates linear IgG and C3 deposition along the dermal-epidermal junction, and circulating antibodies against BP180/BP230 may be detected serologically (25). Although bullous pemphigoid may be idiopathic, it can be triggered by medications such as antibiotics, NSAIDs, diuretics, or dipeptidyl peptidase-4 (DDP4) inhibitors—often becoming apparent after discontinuation of the offending agent (26). The mechanism of immune-related bullous pemphigoid remains unclear. Although sintilimab-associated bullous pemphigoid has been reported in MSS CRC, a definitive association between cutaneous irAEs and antitumor efficacy has not been established (21). In the present case, bullous pemphigoid developed after 19 months of immunotherapy. The patient had type 2 diabetes managed with long-term insulin and no history of DPP-4 inhibitor use. Given that fruquintinib’s main toxicities are hypertension, hand-foot syndrome, and proteinuria—and that bullous pemphigoid has not been associated with this agent—we attribute the reaction to sintilimab. Most immunotherapy-related cutaneous toxicities are grade 1-2, with grade 3–4 being rare; hormones are the mainstay of treatment. This patient experienced grade 2 bullous pemphigoid, which improved significantly after two months of hormone therapy. To preserve antitumor benefit, immunotherapy was cautiously resumed after thorough discussion with the patient.

In this case, the patient achieved sustained disease control of more than 38 months with third-line immunotherapy combined with targeted therapy—far exceeding the median PFS reported in clinical studies—while maintaining a good quality of life. Such outcomes remain rare in the current literature. We speculate that immune-related cutaneous adverse reactions in colorectal cancer may serve as a clinical indicator of response to immunotherapy. The combination of immunotherapy and anti-angiogenic targeted therapy may therefore offer a promising treatment option for patients with pMMR/MSS mCRC, particularly those who are unresponsive to conventional therapies. This case also raises several important considerations. For instance, in patients who maintain NED for only a short period after surgery, it remains unclear whether this is related to underlying genetic factors in the absence of comprehensive molecular testing. While pMMR/MSS tumors are generally regarded as poorly responsive to immunotherapy, the survival achieved in this patient substantially exceeded the expected outcomes of targeted monotherapy and even surpassed the reported efficacy of immunotherapy in deficient mismatch repair (dMMR) CRC. This suggests that beyond MSI/MMR status, additional, as-yet-unidentified biomarkers may be important determinants of immune response and tumor immune escape mechanisms.

Emerging evidence suggests that TMB may be a potential biomarker for predicting ICI responsiveness in patients with pMMR/MSS CRC. In the phase I REGONIVO trial, pMMR patients treated with regorafenib plus nivolumab demonstrated longer median PFS in the high-TMB subgroup (cutoff 22.5 mut/Mb) than in the low-TMB subgroup (12.5 vs. 7.9 months) (10). Similarly, in the CCTG CO.26 trial, MSS CRC patients with high TMB (cutoff 28 mut/Mb) treated with tislelizumab plus durvalumab achieved superior OS compared with those with low TMB (27). In the AtezoTRIBE study, pMMR mCRC patients with high TMB (cutoff 10 mut/Mb) who received atezolizumab in addition to standard therapy experienced increased PFS and OS compared to low-TMB patients (28). However, an optimal cutoff value for TMB in pMMR CRC has not been established, and variability in TMB determination across tumor tissues and individuals highlights the need for further investigation. The level of PD-L1 expression in tumor tissues has also been explored as a potential biomarker of benefit from ICIs (29). Some researchers have also conducted certain explorations on the correlation between PD-L1 expression and the efficacy of ICI treatment in pMMR CRC. In the VOLTAGE-A1 study, neoadjuvant chemoradiotherapy followed by nivolumab in MSS locally advanced rectal cancer showed a higher pathological complete response (pCR) rate in the PD-L1-positive tumor proportion score (TPS) group than in the negative group (75% vs. 17%) (30). A phase II clinical trial evaluating neoadjuvant chemotherapy plus chemoradiotherapy combined with sintilimab found that PD-L1 combined positive score (CPS) correlated with clinical response: the complete response (CR) rate was 53.8% in patients with CPS ≥ 2 versus 28.0% in those with CPS < 2. Tumors with CPS ≥ 2 exhibited enrichment of immune-related pathways (e.g., inflammatory response, IL6/JAK/STAT3, NF-κB, IL2/STAT5) and increased immune cell infiltration (31). These findings suggest PD-L1 may serve as a dynamic biomarker in pMMR CRC. In addition, POLE and POLD1 mutations are associated with defective DNA repair, resulting in hypermutated phenotypes characterized by high TMB and increased tumor-infiltrating lymphocytes (TILs). These attributes support their role as predictive biomarkers for immunotherapy responsiveness (32). A large pan-cancer cohort study identified POLE/POLD1 mutations as independent predictors of ICI response across solid tumors (33), with affected patients demonstrating higher ORR and improved survival (34). Notably, Wen et al. reported four CRC patients with liver metastases harboring pathogenic POLE/POLD1 mutations who achieved pCR following immunotherapy combined with chemotherapy and targeted therapy (35). Furthermore, recent studies suggest that circulating tumor DNA (ctDNA) dynamics and gut microbiota profiles may also correlate with ICI efficacy in pMMR/MSS CRC (36–39).

There are some limitations in this case series. First, the patient was unable to undergo comprehensive genetic testing due to financial constraints, which prevented further identification of potential biomarkers. Continued monitoring will be conducted to help elucidate the potential mechanisms underlying the benefit of immunotherapy in pMMR CRC. Second, the sample size was indeed too small to draw definitive conclusions. Large-scale prospective studies with long-term follow-up are urgently needed to validate our findings.

In summary, this case demonstrates the potential for long-term survival in patients with pMMR mCRC receiving multidisciplinary comprehensive treatment, particularly adjuvant therapy combining immunotherapy and targeted agents. It can be reasonably hypothesized that the patient’s favorable response to immunotherapy may be associated with immunotherapy-related adverse effects or unidentified biomarkers. Overall, this pMMR mCRC patient achieved remarkable efficacy with acceptable safety under the combined regimen of fruquintinib and sintilimab. However, several clinically important questions remain. For instance, the patient has received third-line immunotherapy for over two years—should maintenance therapy be continued? What are the optimal dosing intervals and duration for maintenance immunotherapy? Furthermore, if the patient attains a NED status, can immunotherapy and targeted therapy be safely discontinued? These unresolved issues highlight the need for future clinical studies to further validate the broader applicability of this treatment approach and explore potential predictive biomarkers.

## Data Availability

The original contributions presented in the study are included in the article/supplementary material. Further inquiries can be directed to the corresponding author.

## References

[1] Bray F Laversanne M Sung H Ferlay J Siegel RL Soerjomataram I. Global cancer statistics 2022: GLOBOCAN estimates of incidence and mortality worldwide for 36 cancers in 185 countries. CA: Cancer J Clin. (2024) 74:229–63. doi: 10.3322/caac.21834, PMID: 38572751

[2] Engstrand J Nilsson H Strömberg C Jonas E Freedman J. Colorectal cancer liver metastases - a population-based study on incidence, management and survival. BMC Cancer. (2018) 18:78. doi: 10.1186/s12885-017-3925-x, PMID: 29334918 PMC5769309

[3] Zhou H Liu Z Wang Y Wen X Amador EH Yuan L. Colorectal liver metastasis: molecular mechanism and interventional therapy. Signal Transduct Targeted Ther. (2022) 7:70. doi: 10.1038/s41392-022-00922-2, PMID: 35246503 PMC8897452

[4] Chinese College Of Surgeons CMDA. Guidelines for the diagnosis and comprehensive treatment of liver metastasis from colorectal cancer in China (2023). Chin Clin Med. (2023) 30:166–98.

[5] Li J Qin S Xu R Yau TC Ma B Pan H. Regorafenib plus best supportive care versus placebo plus best supportive care in Asian patients with previously treated metastatic colorectal cancer (CONCUR): a randomised, double-blind, placebo-controlled, phase 3 trial. Lancet Oncol. (2015) 16:619–29. doi: 10.1016/S1470-2045(15)70156-7, PMID: 25981818

[6] Li J Qin S Xu RH Shen L Xu J Bai Y. Effect of fruquintinib vs placebo on overall survival in patients with previously treated metastatic colorectal cancer: the FRESCO randomized clinical trial. Jama. (2018) 319:2486–96. doi: 10.1001/jama.2018.7855, PMID: 29946728 PMC6583690

[7] Xu J Kim TW Shen L Sriuranpong V Pan H Xu R. Results of a randomized, double-blind, placebo-controlled, phase III trial of trifluridine/tipiracil (TAS-102) monotherapy in asian patients with previously treated metastatic colorectal cancer: the TERRA study. J Clin Oncol: Off J Am Soc Clin Oncol. (2018) 36:350–8. doi: 10.1200/JCO.2017.74.3245, PMID: 29215955

[8] Prager GW Taieb J Fakih M Ciardiello F Van Cutsem E Elez E. Trifluridine-tipiracil and bevacizumab in refractory metastatic colorectal cancer. New Engl J Med. (2023) 388:1657–67. doi: 10.1056/NEJMoa2214963, PMID: 37133585

[9] Wang F Jin Y Wang M Luo HY Fang WJ Wang YN. Combined anti-PD-1, HDAC inhibitor and anti-VEGF for MSS/pMMR colorectal cancer: a randomized phase 2 trial. Nat Med. (2024) 30:1035–43. doi: 10.1038/s41591-024-02813-1, PMID: 38438735

[10] Fukuoka S Hara H Takahashi N Kojima T Kawazoe A Asayama M. Regorafenib plus nivolumab in patients with advanced gastric or colorectal cancer: an open-label, dose-escalation, and dose-expansion phase ib trial (REGONIVO, EPOC1603). J Clin Oncol: Off J Am Soc Clin Oncol. (2020) 38:2053–61. doi: 10.1200/JCO.19.03296, PMID: 32343640

[11] Gou M Qian N Zhang Y Yan H Si H Wang Z. Fruquintinib in combination with PD-1 inhibitors in patients with refractory non-MSI-H/pMMR metastatic colorectal cancer: A real-world study in China. Front Oncol. (2022) 12:851756. doi: 10.3389/fonc.2022.851756, PMID: 35875064 PMC9300867

[12] Guo Y Zhang W Ying J Zhang Y Pan Y Qiu W. Phase 1b/2 trial of fruquintinib plus sintilimab in treating advanced solid tumours: The dose-escalation and metastatic colorectal cancer cohort in the dose-expansion phases. Eur J Cancer (Oxford England: 1990). (2023) 181:26–37. doi: 10.1016/j.ejca.2022.12.004, PMID: 36628898

[13] Postow MA Sidlow R Hellmann MD. Immune-related adverse events associated with immune checkpoint blockade. New Engl J Med. (2018) 378:158–68. doi: 10.1056/NEJMra1703481, PMID: 29320654

[14] Brahmer JR Lacchetti C Schneider BJ Atkins MB Brassil KJ Caterino JM. Management of immune-related adverse events in patients treated with immune checkpoint inhibitor therapy: american society of clinical oncology clinical practice guideline. J Clin Oncol: Off J Am Soc Clin Oncol. (2018) 36:1714–68. doi: 10.1200/JCO.2017.77.6385, PMID: 29442540 PMC6481621

[15] Haanen J Obeid M Spain L Carbonnel F Wang Y Robert C. Management of toxicities from immunotherapy: ESMO Clinical Practice Guideline for diagnosis, treatment and follow-up. Ann Oncol: Off J Eur Soc Med Oncol. (2022) 33:1217–38. doi: 10.1016/j.annonc.2022.10.001, PMID: 36270461

[16] Weber JS Hodi FS Wolchok JD Topalian SL SChadendorf D Larkin J. Safety profile of nivolumab monotherapy: A pooled analysis of patients with advanced melanoma. J Clin Oncol: Off J Am Soc Clin Oncol. (2017) 35:785–92. doi: 10.1200/JCO.2015.66.1389, PMID: 28068177

[17] Hofmann L Forschner A Loquai C Goldinger SM Zimmer L Ugurel S. Cutaneous, gastrointestinal, hepatic, endocrine, and renal side-effects of anti-PD-1 therapy. Eur J Cancer (Oxford England: 1990). (2016) 60:190–209. doi: 10.1016/j.ejca.2016.02.025, PMID: 27085692

[18] Hua C Boussemart L Mateus C Routier E Boutros C Cazenave H. Association of vitiligo with tumor response in patients with metastatic melanoma treated with pembrolizumab. JAMA Dermatol. (2016) 152:45–51. doi: 10.1001/jamadermatol.2015.2707, PMID: 26501224

[19] Billon E Walz J Brunelle S Thomassin J Salem N Guerin M. Vitiligo adverse event observed in a patient with durable complete response after nivolumab for metastatic renal cell carcinoma. Front Oncol. (2019) 9:1033. doi: 10.3389/fonc.2019.01033, PMID: 31649889 PMC6795279

[20] Uenami T Hosono Y Ishijima M Kanazu M Akazawa Y Yano Y. Vitiligo in a patient with lung adenocarcinoma treated with nivolumab: A case report. Lung Cancer (Amsterdam Netherlands). (2017) 109:42–4. doi: 10.1016/j.lungcan.2017.04.019, PMID: 28577948

[21] Wang T Shao Q Xiao C Liu L. Case report: Bullous pemphigoid associated with sintilimab therapy for pMMR/MSS colorectal cancer. Front Oncol. (2023) 13:1124730. doi: 10.3389/fonc.2023.1124730, PMID: 36998454 PMC10043161

[22] Mari R Guerin M Vicier C Walz J Bonnet N Pignot G. Durable disease control and refractory bullous pemphigoid after immune checkpoint inhibitor discontinuation in metastatic renal cell carcinoma: A case report. Front Immunol. (2022) 13:984132. doi: 10.3389/fimmu.2022.984132, PMID: 36189265 PMC9524245

[23] Rofe O Bar-Sela G Keidar Z Sezin T Sadik CD Bergman R. Severe bullous pemphigoid associated with pembrolizumab therapy for metastatic melanoma with complete regression. Clin Exp Dermatol. (2017) 42:309–12. doi: 10.1111/ced.13042, PMID: 28211077

[24] Naidoo J Schindler K Querfeld C Busam K Cunningham J Page DB. Autoimmune bullous skin disorders with immune checkpoint inhibitors targeting PD-1 and PD-L1. Cancer Immunol Res. (2016) 4:383–9. doi: 10.1158/2326-6066.CIR-15-0123, PMID: 26928461 PMC5241697

[25] Merli M Accorinti M Romagnuolo M Marzano A Di Zenzo G Moro F. Autoimmune bullous dermatoses in cancer patients treated by immunotherapy: a literature review and Italian multicentric experience. Front Med (Lausanne). (2023) 10:1208418. doi: 10.3389/fmed.2023.1208418, PMID: 37547602 PMC10400335

[26] Vassileva S. Drug-induced pemphigoid: bullous and cicatricial. Clinics Dermatol. (1998) 16:379–87. doi: 10.1016/S0738-081X(98)00008-X, PMID: 9642531

[27] Chen EX Jonker DJ Loree JM Kennecke HF Berry SR Couture F. Effect of combined immune checkpoint inhibition vs best supportive care alone in patients with advanced colorectal cancer: the canadian cancer trials group CO.26 Study. JAMA Oncol. (2020) 6:831–8. doi: 10.1001/jamaoncol.2020.0910, PMID: 32379280 PMC7206536

[28] Antoniotti C Rossini D Pietrantonio F Salvatore L Lonardi S Tamberi S. Upfront fluorouracil, leucovorin, oxaliplatin, and irinotecan plus bevacizumab with or without atezolizumab for patients with metastatic colorectal cancer: updated and overall survival results of the ATEZOTRIBE study. J Clin Oncol: Off J Am Soc Clin Oncol. (2024) 42:2637–44. doi: 10.1200/JCO.23.02728, PMID: 38865678

[29] Davis AA Patel VG. The role of PD-L1 expression as a predictive biomarker: an analysis of all US Food and Drug Administration (FDA) approvals of immune checkpoint inhibitors. J Immunother Cancer. (2019) 7:278. doi: 10.1186/s40425-019-0768-9, PMID: 31655605 PMC6815032

[30] Bando H Tsukada Y Inamori K Togashi Y Koyama S Kotani D. Preoperative Chemoradiotherapy plus Nivolumab before Surgery in Patients with Microsatellite Stable and Microsatellite Instability-High Locally Advanced Rectal Cancer. Clin Cancer Res: Off J Am Assoc Cancer Res. (2022) 28:1136–46. doi: 10.1158/1078-0432.CCR-21-3213, PMID: 35063964 PMC9365382

[31] Xiao WW Chen G Gao YH Lin JZ Wu XJ Luo HL. Effect of neoadjuvant chemoradiotherapy with or without PD-1 antibody sintilimab in pMMR locally advanced rectal cancer: A randomized clinical trial. Cancer Cell. (2024) 42:1570–81.e4. doi: 10.1016/j.ccell.2024.07.004, PMID: 39094560

[32] Carvajal-Veloza J Galindo-Morales F Gutierrez-Castañeda LD. Functions, interactions and prognostic role of POLE: a bioinformatics analysis. J Gynecol Oncol. (2025) 36:e45. doi: 10.3802/jgo.2025.36.e45, PMID: 39575998 PMC12099036

[33] Wang F Zhao Q Wang YN Jin Y He MM Liu ZX. Evaluation of POLE and POLD1 mutations as biomarkers for immunotherapy outcomes across multiple cancer types. JAMA Oncol. (2019) 5:1504–6. doi: 10.1001/jamaoncol.2019.2963, PMID: 31415061 PMC6696731

[34] Nunes L Li F Wu M Luo T Hammarström K Torell E. Prognostic genome and transcriptome signatures in colorectal cancers. Nature. (2024) 633:137–46. doi: 10.1038/s41586-024-07769-3, PMID: 39112715 PMC11374687

[35] Wen L Chen Z Ji X Fong WP Shao Q Ren C. Pathological complete response to immune checkpoint inhibitor in patients with colorectal cancer liver metastases harboring POLE exonuclease domain mutation. J Immunother Cancer. (2022) 10(7):e004487. doi: 10.1136/jitc-2022-004487, PMID: 35793867 PMC9260839

[36] Kasi PM Hidalgo M Jafari MD Yeo H Lowenfeld L Khan U. Neoadjuvant botensilimab plus balstilimab response pattern in locally advanced mismatch repair proficient colorectal cancer. Oncogene. (2023) 42:3252–9. doi: 10.1038/s41388-023-02835-y, PMID: 37731056 PMC10611560

[37] Zhao W Lei J Ke S Chen Y Xiao J Tang Z. Fecal microbiota transplantation plus tislelizumab and fruquintinib in refractory microsatellite stable metastatic colorectal cancer: an open-label, single-arm, phase II trial (RENMIN-215). EClinicalMedicine. (2023) 66:102315. doi: 10.1016/j.eclinm.2023.102315, PMID: 38024475 PMC10679864

[38] Williams CJM Peddle AM Kasi PM Seligmann JF Roxburgh CS Middleton GW. Neoadjuvant immunotherapy for dMMR and pMMR colorectal cancers: therapeutic strategies and putative biomarkers of response. Nat Rev Clin Oncol. (2024) 21:839–51. doi: 10.1038/s41571-024-00943-6, PMID: 39317818

[39] Mager LF Burkhard R Pett N Cooke NCA Brown K Ramay H. Microbiome-derived inosine modulates response to checkpoint inhibitor immunotherapy. Sci (New York NY). (2020) 369:1481–9. doi: 10.1126/science.abc3421, PMID: 32792462

